# Comparative evaluation of plasma and serum HIV-1 viral load measurements among HIV positive individuals, Northwest Ethiopia: Analytical cross-sectional study

**DOI:** 10.1371/journal.pone.0315717

**Published:** 2025-03-03

**Authors:** Getu Girmay, Habtam Alemu, Muluneh Assefa, Nega Birhane, Mulualem Lemma

**Affiliations:** 1 Department of Immunology and Molecular Biology, School of Biomedical and Laboratory Science, College of Medicine and Health Sciences, University of Gondar, Gondar, Ethiopia; 2 University of Gondar Comprehensive Specialized Hospital, Gondar, Ethiopia; 3 Department of Medical Microbiology, School of Biomedical and Laboratory Science, College of Medicine and Health Sciences, University of Gondar, Gondar, Ethiopia; 4 Department of Medical Biotechnology, Institute of Biotechnology, University of Gondar, Gondar, Ethiopia; Kwame Nkrumah University of Science and Technology, GHANA

## Abstract

**Introduction:**

Plasma HIV viral load tests have been widely used in clinical practice to monitor treatment success or failure. Inappropriate mixing with anticoagulants and inaccessibility of plasma samples in certain clinical services (such as antenatal care services) might hinder HIV diagnosis and treatment services. Considering that serum has higher stability and availability in prenatal care services, periodic monitoring of serum HIV viral load might be an alternative approach. Thus, this study aimed to evaluate the plasma and serum HIV-1 viral load measurements among HIV-positive individuals in Northwest Ethiopia.

**Methods:**

An institution-based analytical cross-sectional study was conducted on 74 paired plasma and serum samples from May to August 2020 at the HIV Treatment Center, Northwest Ethiopia. Four milliliters of paired venous blood were collected to harvest plasma and serum. HIV-1 RNA was extracted and quantified using the Roche COBAS AmpliPrep/COBAS TaqMan assay. Data were analyzed using SPSS version 20. Paired sample *t*-tests and Pearson’s correlation were employed to observe the mean differences and associations between sample measurements, respectively. Bland–Altman and linear regression models were computed to demonstrate the level of agreement and proportional bias, respectively. A p-value of ≤  0.05 with a 95% confidence interval was considered statistically significant.

**Results:**

From a total of 74 HIV-positive individuals, 49 (66.2%) were females. The mean ±  SD age was 36.3 (10.1) years. The mean difference ±  SD of plasma and serum HIV-1 viral load was 0.07 ±  0.75 log copies/mL, (*p* =  0.428). A strong association with a significant linear correlation (r =  0.862) (*p* < 0.001) and a high level of agreement were observed between the sample measurements using Pearson’s correlation and a Bland–Altman plot, respectively.

**Conclusions:**

The current study highlights an alternative application of serum-based HIV viral load quantification to enhance the rate of HIV diagnosis and treatment monitoring coverage.

## Introduction

Human immunodeficiency virus (HIV) continues to have a significant public health impact, especially in developing countries [1]. As per the World Health Organization (WHO) 2022 estimate, there were around 39 million individuals worldwide living with HIV, and two-thirds of those individuals were living in the WHO’s African regions [2,3]. HIV viral load testing is utilized for the diagnosis of primary HIV infection, which is a therapeutic emergency due to the extremely high viremia during this phase of illness. According to current guidelines, highly active antiretroviral therapy (HAART) should be initiated as soon as feasible to reduce the size of the HIV reservoir [4,5]. Likewise, following six months of HIV infection, the quantity of viral ribonucleic acid (RNA) that has been established is highly predictive of the progression of HIV infection [6].

The World Health Organization has set a goal where, by 2030, 95% of all HIV-positive individuals will have received a diagnosis, 95% of them will be receiving life-saving antiretroviral treatment, and 95% of those receiving treatment will have achieved a suppressed viral load to reduce the transmission rate of HIV infection [7,8]. A comprehensive diagnosis of HIV infection is attained through the quantification of HIV viral RNA, which is one of the strongest predictors of disease progression and an indicator of treatment success or failure [9,10]. Plasma HIV viral load measurements have been routinely utilized in clinical practice, and it is still crucial to periodically evaluate the plasma HIV-1 viral load to monitor HIV-positive individuals receiving antiretroviral therapy (ART) [11,12]. Evidence has shown that HIV-1 viral load levels below 20 to 40 copies/mL are suggestive of a successful outcome for combined antiretroviral therapy, which reduces the HIV infection transmission rate in those who are vulnerable groups [13,14].

It is essential to increase the availability of HIV viral load measurements to meet the WHO’s 2030 goal and improve the rate of viral suppression among HIV-positive individuals receiving ART [7,15]. HIV diagnosis and treatment coverage, however, might be hindered by test interference due to improper mixing of blood and anticoagulants during plasma sample processing, as well as by certain challenging circumstances such as if a patient is using prenatal care services during pregnancy (serum is the most commonly available test sample), particularly in resource-limited settings [16,17]. Thus, serum might be a suitable alternative sample for the periodic monitoring of HIV viral load to assess the initiation or efficacy of ART regimens, due to its higher stability and increased accessibility in patients under antenatal care services. Thus, this study aimed to evaluate the HIV-1 viral load measurements in plasma and serum samples by using the Roche COBAS AmpliPrep/COBAS TaqMan version 2.0 assay among HIV-1 positive individuals at the University of Gondar Comprehensive Specialized Hospital’s HIV Treatment Center, Gondar, Northwest Ethiopia.

## Materials and Methods

### Study design, period, and setting

An institution-based analytical cross-sectional study was conducted among HIV-positive individuals, at the University of Gondar Comprehensive Specialized Hospital (UoG-CSH)’s HIV Treatment Center, Gondar, Northwest Ethiopia, from 17 May to 12 August 2020. The UoG-CSH is located in the Central Gondar Zone of the Amhara Regional state, 747 km from Addis Ababa (the capital city of Ethiopia). In Gondar town, there are around eight health centers, two private hospitals, around 42 private clinics, and one comprehensive specialized and referral hospital (UoG-CSH). The UoG-CSH is a teaching hospital that offers comprehensive specialty and referral services to more than seven million individuals who live in Gondar town and the central Gondar zone, as well as people in the neighboring zones. Currently, the UoG-CSH provides services to around 5584 HIV-positive individuals at the UoG-CSH HIV Treatment Center.

### Source and study populations

All HIV-positive individuals who were receiving ART at the UoG-CSH HIV Treatment Center were considered our source population. The study population included HIV patients of any age who underwent ART for at least six months at the UoG-CSH HIV Treatment Center and were available during the study period. Individuals who were critically ill and unable to give blood samples and HIV-positive individuals who had undergone ART for less than six months were excluded from the study.

### Sample size determination and sampling procedure

The sample size was calculated using the G-power software, version 3.1.9.7 [18]. Using a paired sample *t*-test approach aimed to determine the mean difference between two matched pairs, medium effect size (d =  0.3), 5% significant level (α =  0.05), and 80% of desired power (1-β =  0.80). Considering a 5% non-response rate, the ultimate sample size obtained was 74. A simple random sampling technique using lottery methods was applied to recruit on ART HIV-positive individuals who visited the UoG-CSH HIV Treatment Center from May to August 2020, until the ultimate sample size was attained.

### Operational definition

**Adherence**: the level to which HIV-positive individuals who are undergoing ART treatment comply with their treatment regimen.

**Good adherence**: 95% adherence to medication, or less than 2 missed doses of 30 doses, or less than 3 missed doses of 60 doses [19,20].

**Fair adherence**: 85–94% drug adherence, or 3–5 missed drug doses of 30, or 4–5 missed drug doses of 60 doses [19,20].

**Poor adherence**: less than 85% drug adherence, or at least 6 missed doses of 30 ART drug doses, or more than 9 missed doses of 60 ART drug doses [19,20].

### Data collection

Sociodemographic and clinical data of the study participants were collected from patient’s charts and medical records, using a structured data collection form, by a well-trained clinical nurse who worked in the UoG-CSH HIV treatment center (S1 Table).

### Blood sample collection and processing

Four milliliters (4 mL) of venous blood was collected from HIV-positive individuals using vacutainer tubes containing EDTA anti-coagulant and a serum separator tube (SST) for plasma and serum HIV-1 viral load determination, respectively, by an experienced laboratory technologist. The blood samples were transported to the UoG-CSH ART laboratory within four hours of collection. Plasma and serum were harvested from whole blood samples through centrifugation for 10 minutes at 2500 revolutions per minute (RPM). Then, aliquots of plasma and serum samples were prepared in the cryovials and stored at − 80°C until they were tested for HIV-1 viral load.

### HIV-1 RNA extraction from plasma and serum samples

An aliquot containing 1100 µ L of plasma, serum, negative control, HIV-1 low positive control, and HIV-1 high positive control samples were prepared and loaded into the COBAS AmpliPrep instrument (Roche Molecular Systems, Inc., Branchburg, NJ, USA) for automated specimen processing. The HIV-1 RNA was extracted from 1100 µ L of plasma and serum samples using the silica-based capture method. Protease combined with chaotropic lysis solution was used to lyse the HIV-1 viral particles. In addition, a known concentration of HIV-1 Quantitation Standard (QS) RNA molecules (Ambion, Inc. and Cenetron Diagnostics, LLC., Branchburg, NJ, USA) was added to each plasma and serum sample. The target HIV-1 RNA and HIV-1 QS RNA were then bound to the magnetic glass particle surface, and unbound substances and impurities were removed through washing [14,21].

### HIV-1 viral load quantification

Following automated specimen processing, the target HIV-1 RNA and HIV-1 QS RNA were added to an amplification mixture containing buffer solution, manganese (Mn^2+^), and deoxynucleotide triphosphates (dNTPs), and then loaded onto the COBAS TaqMan version 2.0 Analyzer (Roche Molecular Systems, Inc., Branchburg, NJ, USA) for reverse transcription, amplification, and detection. Thermus species DNA polymerase, a thermostable recombinant enzyme, was used for reverse transcription and amplification of the target HIV-1 RNA and HIV-1 QS RNA. The target HIV-1 RNA and HIV-1 QS RNA were denatured at 94°C. Subsequently, primer annealing and DNA extension were employed at 54°C and 72°C, respectively, to produce a double-stranded DNA molecule. Detection of the target HIV-1 RNA and HIV-1 QS RNA was done using HIV-1- and HIV-1 QS-specific oligonucleotide probes labeled with fluorescent dye. The fluorescent signals were interpreted using AMPLILINK version 3.4 software. The negative control, HIV-1 low positive control, and HIV-1 high positive control results were interpreted as a target not detected (<20 copies/mL), 20–726 copies/mL, and 727–312,688 copies/mL, respectively. In addition, the plasma and serum HIV-1 viral load measurements were reported as a target not detected and as being within the quantitative range of 20–10^7^ copies/mL [14,21].

### Data management and quality control

Relevant clinical data and blood samples were collected by a trained clinical nurse and an experienced laboratory technologist, respectively. Standard operating protocols were followed for sample collection, processing, and laboratory testing. All assays were performed according to the manufacturer’s instructions and following the standard operational procedures during each assay test. The quality of HIV-1 RNA sample processing was assessed by incorporating negative, HIV-1 low positive, and HIV-1 high positive controls along with the patient samples. The laboratory processing was performed under a controlled laboratory environment using a class II biosafety cabinet and strictly adhered to the laboratory safety guidelines to ensure the reliability and precision of the results [22,23].

### Data analysis

Data were entered, cleaned, and analyzed using SPSS version 20 software. Descriptive statistics such as means and cross-tabulation tests were computed to characterize the study participants and compare quantitative HIV-1 viral load results. Paired sample *t*-tests were conducted to determine the mean difference between plasma and serum sample measurements. Pearson’s correlation analysis was applied to compare the associations between sample measurements. The level of agreement between sample measurements and the proportional bias were evaluated using Bland–Altman analysis and linear regression models, respectively. A *p*-value of ≤ 0.05 with a 95% confidence interval was considered statistically significant.

### Ethical statement

Ethical approval was obtained from the ethics committee of the Institute of Biotechnology, University of Gondar, Gondar, Ethiopia, with the reference number IOB/910/05/2020. All study participants provided written informed consent, and written informed assent was also obtained from the parents/guardians of study participants under 18 years of age. A support letter was obtained from the UoG-CSH medical director. A unique code was assigned to each study participants to ensure their confidentiality, and the study was conducted as per the Helsinki Declaration for Biomedical Research [24,25].

## Results

### Socio-demographic and clinical characteristics of study participants

A total of 74 HIV-positive individuals were enrolled in this study to collect paired plasma and serum samples. Out of these, 49 (66.2%) were female and 25 (33.8%) were male. The mean ±  standard deviation (SD) age was 36.3 (10.1) years. The minimum and maximum ages of HIV-positive individuals were 9 and 60 years, respectively. The majority of HIV-positive individuals (52.7%) were above 36 years of age, followed by 18–35 years of age (41.9%). Sixty-nine (93.2%) of the study participants had a stage I HIV clinical classification. Of the included study participants, 89.2% (66) and 95.9% (71) were undergoing a first-line treatment regimen and had fair treatment adherence, respectively (Table 1).

**Table 1:** Sociodemographic and clinical characteristics of HIV-positive individuals at the UoG-CSH, Northwest Ethiopia.

Variables	Category	Frequency (n)	Percentage (%)
Sex	Male	25	33.8
	Female	49	66.2
Age (Years)	< 17	4	5.4
	18-35	31	41.9
	> 36	37	52.7
HIV clinical stage	I	69	93.2
	II	5	6.8
Treatment regimen	First line^a^	66	89.2
	Second line^b^	6	8.1
	Third line^c^	2	2.7
Treatment adherence	Fair	71	95.9
	Poor	1	1.4
	Good	2	2.7

^a^: adults who were treated with ABC + 3TC +  EFV or TDF +  3TC +  DTG and children treated with ABC +  3TC +  LPV/r or ABC +  3TC +  DTG.

^b^: adults who were treated with TDF +  3TC +  LPV/r or TDF +  3TC +  DTG and children treated with ABC +  3TC +  LPV/r or TDF +  3TC +  EFV.

^c^: adults who were treated with DRV/r +  DTG +  TDF +  3TC or DRV/r +  TDF +  3TC +  EFV and children treated with DRV/r +  DTG +  TDF +  3TC or DRV/r +  ABC +  3TC +  EFV.ABC: abacavir, 3TC: lamivudin, EFV: efavirenz, TDF: tenofovir disoproxil fumarate, LPV/r: lopinavir/ritonavir, DRV/r: darunavir/ritonavir, HIV: human immunodeficiency virus.

### Quantitative HIV-1viral load in plasma and serum measurements

Quantitative HIV-1 viral load results were obtained from 74 (100%) paired plasma and serum measurements. Of the included 74 paired plasma and serum samples, 16 (21.6%) and 8 (10.8%) of the plasma HIV-1 viral loads were detected as 20–1000 copies/mL (1.30–3.0 log copies/mL) and > 1000 copies/mL (>3.0 log copies/mL), respectively. From the serum HIV-1 viral load results, 16 (21.6%) and 9 (12.2%) of the samples were detected as 20–1000 copies/mL (1.30–3.0 log copies/mL) and > 1000 copies/mL (>3.0 log copies/mL), respectively. However, 44 (59.5%) of the included paired plasma and serum samples show undetected HIV-1 viral load results, which were below the low detection limit of the Roche COBAS AmpliPrep/COBAS TaqMan assay (<1.30 log copies/mL; 20 copies/mL) (Table 2).

**Table 2:** Quantitative HIV-1 viral load measured by Roche COBAS AmpliPrep/COBAS TaqMan assay in plasma versus serum samples.

Plasma HIV-1 viral load (n = 74)					
**Serum HIV-1 viral load (n = 74)**		Not detected^a^	20–1000 copies/ml^b^	>1000 copies/ml^c^	Total
	Not detected^a^	44	5	0	49
	20–1000 copies/ml^b^	6	10	0	16
	>1000 copies/ml^c^	0	1	8	9
	Total	50	16	8	74

^a^: below the low detection limit, < 20 copies/mL (<1.30 log_10_ copies/mL);

^b^: 20–1000 copies/mL (1.30–3.0 log_10_ copies/mL);

^c^: > 1000 copies/mL (>3.0 log_10_ copies/mL), HIV: human immunodeficiency virus.

### Comparison of quantitative HIV-1 viral load in plasma and serum measurements

**Fig 1:**
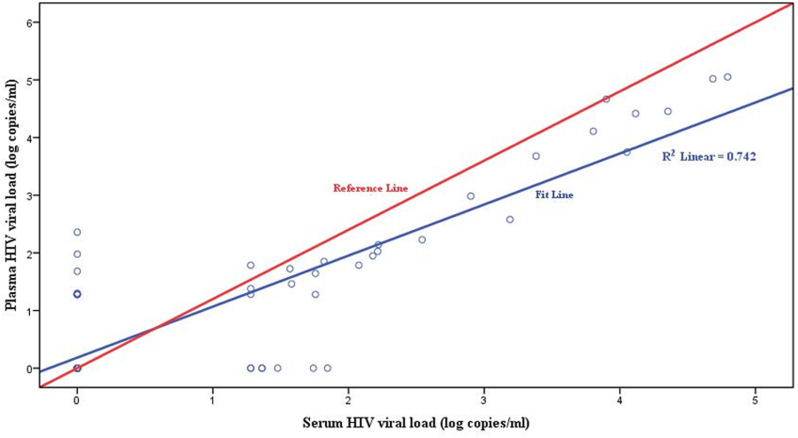
Correlation analysis of HIV-1 viral load (VL) in paired plasma versus serum measurements.

A paired sample *t*-test was used to compare the mean difference between the HIV-1 viral load measurements of the plasma and serum samples. The mean difference with SD between the plasma and serum HIV-1 viral load measurements was 0.07 ±  0.75 log copies/mL, with no statistically significant difference (*p* =  0.428). The association between the plasma and serum HIV-1 viral load measurements was assessed using Pearson’s correlation analysis. The results show that there was a strong correlation between the HIV-1 viral load results, with a significant linear correlation over the quantification range (r =  0.862) of 74 paired plasma and serum samples (*p* < 0.001) (Fig 1).

### Evaluation of the agreement between plasma and serum HIV-1 viral load measurements

**Fig 2:**
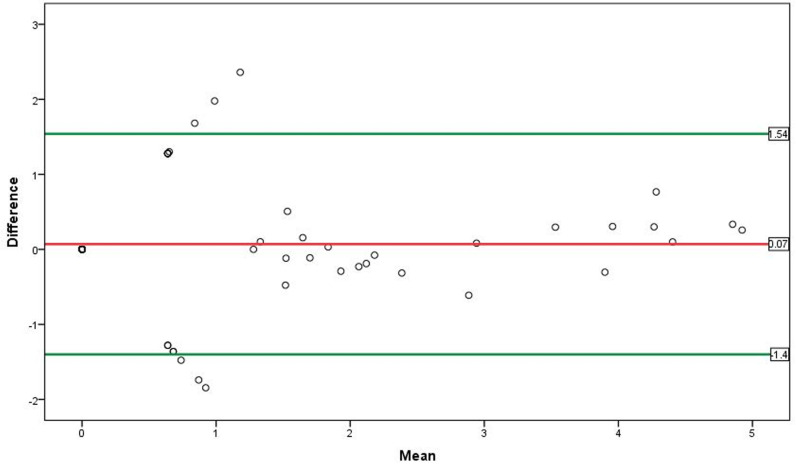
Bland–Altman plot comparing HIV-1 viral loads (VLs) between paired plasma and serum measurements.

Following a paired sample *t*-test, we found a non-significant mean difference (*p* =  0.428) between plasma and serum HIV-1 viral load measurements. Thus, we used the Bland–Altman test to compute the level of agreement between those measurements. The difference plot for the HIV-1 viral load results showed that the mean difference was 0.07 (95% CI; − 1.40–1.54) log copies/mL between the plasma and serum measurements. As depicted in the Bland–Altman plot, there was a high level of agreement between the plasma and serum HIV-1 viral load measurements (Fig 2). In addition, no significant proportional bias (*p* =  0.652) was observed following a linear regression analysis with a standardized beta coefficient mean value of 0.053 log copies/mL.

## Discussion

HIV infection continues to have devastating consequences on individuals across the globe [2]. The most efficient approach for managing individuals with HIV is through the quantification of HIV RNA. Therefore, having the capability to carry out viral load testing is crucial for identifying early treatment failure, which improves the success rate of prompt actions and reduces the burden of drug resistance mutations [9,10].

In this study, the Roche COBAS AmpliPrep/COBAS TaqMan version 2.0 assay was used to quantify the HIV-1 viral load results of prospectively collected plasma and serum samples. The findings show that the HIV-1 viral load measurements were highly correlated, with a significant linear correlation between the plasma and serum samples. The mean difference with SD between the plasma and serum measurements was not significantly different when analyzed using a paired sample *t*-test (Fig 1). In the current study, the level of agreement between the plasma and serum measurements in terms of quantitative HIV-1 viral load results was demonstrated using a Bland–Altman plot. The difference plot depicted a high level of agreement between the two paired samples (plasma and serum) for the quantitative detection of HIV-1 viral load among individuals receiving ART, as shown in Fig 2.

Our findings was supported by a previous study from France [26], which demonstrated that HIV-1 viral load measurements analyzed using the Alinity m assay were highly correlated, and no significant mean difference was observed between the plasma and serum samples. In addition, the current findings are further supported by the findings of a previous study from West Haven, Connecticut [27], which demonstrated that, in quantitative HIV-1 viral load results generated using the Amplicor HIV-1 Monitor test, there were no significant quantitative HIV-1 viral load results, and the results were almost comparable across the plasma and serum samples.

The WHO has planning that, by 2030, 95% of all HIV-positive people will have been diagnosed, 95% will be on life-saving antiretroviral therapy, and 95% will have achieved a suppressed viral load to prevent HIV from spreading to others [7,8]. In this context, high correlation and agreement between plasma and serum samples could be particularly important for enhancing HIV viral load detection coverage when using an alternative serum sample, which may be used if a plasma sample is not available (for example, if a patient is under antenatal care services) or due to the higher stability of serum samples. Adopting this alternative approach could potentially enable significant reductions in the turnaround time of the diagnosis and enhance the workflow in the laboratory. Therefore, the lack of significant differences between the plasma and serum quantitative HIV-1 RNA measurements observed in this study might contribute to the application of an alternative serum sample-based approach for the determination of HIV-1 RNA load in areas with limited infrastructure. A limitation of the study is that we could not evaluate the plasma and serum HIV-1 viral load measurements of samples kept in different storage conditions.

## Conclusion and recommendations

In this study, we found a high correlation between the HIV-1 RNA measurements of plasma and serum samples, with a significant linear correlation over the quantitative range. In addition, there was a high level of agreement between the plasma and serum measurements in terms of HIV-1 viral load results, as depicted using a Bland–Altman plot. Thus, to enhance the rate of HIV diagnosis and treatment monitoring coverage, particularly in settings with limited infrastructure, alternative serum-based approaches for HIV-1 viral load quantification can be applicable.

## Supporting information

S1 Checklist STROBE checklist for observational study. (DOCX)

S1 Table Sociodemographic and clinical characteristics assessment questionnaire. (PDF)

## Abbreviations

ART: antiretroviral therapy
HAART: highly active antiretroviral therapy
HIV: human immunodeficiency virus
QS: quantification standard
RNA: ribonucleic acid
UoG-CSH: University of Gondar Comprehensive Specialized Hospital
WHO: World Health Organization
