# Supplementary material for: Comparative evaluation of plasma and serum HIV-1 viral load measurements among HIV positive individuals, Northwest Ethiopia: Analytical cross-sectional study
Source: PLoS One. 2025 Mar 3;20(3):e0315717. doi: 10.1371/journal.pone.0315717 (PMC11875351; doi:10.1371/journal.pone.0315717)
Supplement: S1 Checklist — (DOCX) [file pone.0315717.s001.docx]

S1 Checklist. STROBE checklist for observational study

|  | Item No. | Recommendation | Page No | Relevant text from manuscript |
| --- | --- | --- | --- | --- |
| **Title and abstract** | 1 | (a) Indicate the study’s design with a commonly used term in the title or the abstract | 1 | Analytical cross-sectional study |
|  |  | (b) Provide in the abstract an informative and balanced summary of what was done and what was found | 1-2 |  |
| Introduction | | | |  |
| Background/rationale | 2 | Explain the scientific background and rationale for the investigation being reported | 2-4 |  |
| Objectives | 3 | State specific objectives, including any prespecified hypotheses | 4 |  |
| Methods | | | |  |
| Study design | 4 | Present key elements of study design early in the paper | 4 |  |
| Setting | 5 | Describe the setting, locations, and relevant dates, including periods of recruitment, exposure, follow-up, and data collection | 4 |  |
| Participants | 6 | Cross-sectional study—Give the eligibility criteria, and the sources and methods of selection of participants | 4-5 |  |
| Variables | 7 | Clearly define all outcomes, exposures, predictors, potential confounders, and effect modifiers. Give diagnostic criteria, if applicable |  | NA |
| Data sources/ measurement | 8* | For each variable of interest, give sources of data and details of methods of assessment (measurement). Describe comparability of assessment methods if there is more than one group | 6-8 | Aliquots containing 1100 µl of plasma and serum were prepared, HIV RNA was extracted and quantified using Roche COBAS AmpliPrep/COBAS TaqMan version 2.0 assay |
| Bias | 9 | Describe any efforts to address potential sources of bias |  | NA |
| Study size | 10 | Explain how the study size was arrived at | 5 | Sample size was calculated using the G-power software. Using a paired sample *t*-test aimed to determine the mean difference of two matched pairs, medium effect size (d = 0.3), 5% significant level (α = 0.05), and 95% of desired power (1-β = 0.80). |

| Quantitative variables | 11 | Explain how quantitative variables were handled in the analyses. If applicable, describe which groupings were chosen and why | 6-8 |  |
| --- | --- | --- | --- | --- |
| Statistical methods | 12 | (a) Describe all statistical methods, including those used to control for confounding | 8 |  |
|  |  | (b) Describe any methods used to examine subgroups and interactions |  | NA |
|  |  | (c) Explain how missing data were addressed | 8 |  |
|  |  | (d) Cross-sectional study—If applicable, describe analytical methods taking account of sampling strategy |  | NA. |
|  |  | (e) Describe any sensitivity analyses |  | NA |
| Results | | | | |
| Participants | 13* | (a) Report numbers of individuals at each stage of study—eg numbers potentially eligible, examined for eligibility, confirmed eligible, included in the study, completing follow-up, and analysed | 9 | A total of 74 HIV-positive individuals were enrolled to collect paired plasma and serum samples. The minimum and maximum age of HIV-positive individuals were 9 and 60 years, respectively. |
|  |  | (b) Give reasons for non-participation at each stage |  | NA |
|  |  | (c) Consider use of a flow diagram |  | NA |
| Descriptive data | 14* | (a) Give characteristics of study participants (eg demographic, clinical, social) and information on exposures and potential confounders | 9 |  |
|  |  | (b) Indicate number of participants with missing data for each variable of interest |  | NA |
| Outcome data | 15* | Cross-sectional study—Report numbers of outcome events or summary measures | 9-11 |  |
| Main results | 16 | (a) Give unadjusted estimates and, if applicable, confounder-adjusted estimates and their precision (eg, 95% confidence interval). Make clear which confounders were adjusted for and why they were included | 9-11 |  |
|  |  | (b) Report category boundaries when continuous variables were categorized |  | NA |
|  |  | (c) If relevant, consider translating estimates of relative risk into absolute risk for a meaningful time period |  | NA |

Continued on next page

| Other analyses | 17 | Report other analyses done—eg analyses of subgroups and interactions, and sensitivity analyses |  | NA |
| --- | --- | --- | --- | --- |
| Discussion | | | | |
| Key results | 18 | Summarise key results with reference to study objectives | 11-12 | The findings showed that the HIV viral load measurements were highly correlated with a significant linear correlation between the plasma and serum samples. The difference plot depicted that there was a high level of agreement between the two paired samples (plasma and serum) for the quantitative detection of HIV viral load. |
| Limitations | 19 | Discuss limitations of the study, taking into account sources of potential bias or imprecision. Discuss both direction and magnitude of any potential bias | 12 | We could not evaluate the plasma and serum HIV viral load measurements with different storage conditions. |
| Interpretation | 20 | Give a cautious overall interpretation of results considering objectives, limitations, multiplicity of analyses, results from similar studies, and other relevant evidence | 11-12 |  |
| Generalisability | 21 | Discuss the generalisability (external validity) of the study results | 13 |  |
| Other information | |  | | |
| Funding | 22 | Give the source of funding and the role of the funders for the present study and, if applicable, for the original study on which the present article is based | 14 |  |
