# Supplementary material for: Comparative evaluation of plasma and serum HIV-1 viral load measurements among HIV positive individuals, Northwest Ethiopia: Analytical cross-sectional study
Source: PLoS One. 2025 Mar 3;20(3):e0315717. doi: 10.1371/journal.pone.0315717 (PMC11875351; doi:10.1371/journal.pone.0315717)
Supplement: S1 Table — (PDF) [file pone.0315717.s002.pdf]

S1 Table. Sociodemographic and clinical characteristics assessment questionnaire

No	Variables	Response
**Sociodemographic and clinical characteristics**		
101	Age	_________ years
102	Sex	1. Male 2. Female
103	Reason for HIV viral load appointment	1. Annual 2. First visit 3. Second visit 4. Suspected treatment failure
104	HIV clinical stage	1. I 2. II 3. III 4. IV
105	HIV treatment regimen	_______________
106	HIV treatment adherence	1. Good 2. Fair 3. Poor
107	Plasma HIV-1 viral load	_______________ log copies/ml
108	Serum HIV-1 viral load	_______________ log copies/ml
